# Decision-making capacity and communication about care of older people during their last three months of life

**DOI:** 10.1186/1472-684X-12-1

**Published:** 2013-01-10

**Authors:** Pam J Kaspers, Bregje D Onwuteaka-Philipsen, Dorly JH Deeg, H Roeline W Pasman

**Affiliations:** 1 Department of Public and Occupational Health, and Expertise Centre for Palliative Care Amsterdam, EMGO+ Institute for Health and Care Research, VU University Medical Centre, Van der Boechorststraat 7, 1081, BT, Amsterdam, The Netherlands; 2 Department of Epidemiology and Biostatistics, LASA, EMGO+ Institute for Health and Care Research, VU University Medical Centre, Van der Boechorststraat 7, 1081, BT, Amsterdam, The Netherlands

**Keywords:** Decision-making capacity, End-of-life care, Older people, Advance directives, Appointed proxy

## Abstract

**Background:**

Limited decision-making capacity (DMC) of older people affects their abilities to communicate about their preferences regarding end-of-life care. In an advance directive (AD) people can write down preferences for (non)treatment or appoint a proxy as a representative in (non)treatment choices in case of limited DMC.

The aim is to study limited DMC during the end of life and compare the background, (satisfaction with) care and communication characteristics of people with and without limited DMC. Furthermore, the aim is to describe patient proxies’ opinions about experiences with the use of (appointed proxy) ADs.

**Methods:**

Using a questionnaire, data were collected from proxies of participants of a representative sample of the Longitudinal Aging Study Amsterdam (n=168) and a purposive sample of the Advance Directive cohort study (n=184). Differences between groups (with and without limited DMC, and/or with and without AD) were tested with chi-square tests, using a level of significance of p < 0.05.

**Results:**

At a month before death 27% of people had limited DMC; this increased to 67% of people having limited DMC in the last week of life. The care received was in accordance with the patient’s preferences for the majority of older people, although less often for people who had limited DMC for more than a week. The majority of the proxies were satisfied with the communication between physician and the patient and them, regardless of DMC of the patient. Of people with an AD, a small majority of relatives indicated that the AD had been of additional value. Finally, no differences were found in the role of the relative and the satisfaction with this role between people with and without a proxy AD.

**Conclusions:**

Although relatives have positive experiences with ADs, our study does not provide strong evidence that (proxy) ADs are very influential in the last phase of life. They can best be seen as a tool for advance care planning.

## Background

Older people are frequently affected by multiple progressive illnesses, many of which arise during the last year of life [1]. In the course of these illnesses older people may lose the capacity to accept or reject medical treatments. Limited decision-making capacity (DMC) can occur in degrees and last for a longer or shorter time period and can have various causes, e.g. due to a progression of dementia, reduced consciousness or (sub)comatose state due to a physical illness. Limitations in DMC of older people affect their abilities to communicate about their care preferences in end-of-life decisions. Decision-making capacity has been divided into five dimensions: understanding the facts involved in the decision, appreciation of the nature and importance of the decision, understanding the benefits and risks of the decision, communication about the decision, and deliberation based on personal values [2,3].

As patients’ quality of life is a primary focus in end-of-life care it is important that others are familiar with their preferences when they become unable to express those preferences themselves. People can prepare for such a situation by discussing their preferences with both health care professionals and proxies (advance care planning). A comparison between patients with usual care plus advance care planning and patients with only usual care showed that advance care planning improved end-of-life care and patient and family satisfaction [4]. People may write down their preferences in an advance directive (AD), a written statement in which they can specify preferences in end-of-life care and treatment decisions. An AD becomes of relevance when a person loses his or her capacity to make decisions. An AD can either make statements about receiving or refusing treatments in certain situations or a statement in which the person can appoint a health care proxy who will represent the individual when he or she has limited DMC. In the Netherlands, ADs that concern refusal of treatment are legally binding.

Previous studies on DMC at the end of life have predominantly explored the terminology, how to assess DMC, hypothetical situations of limited DMC, DMC and end-of-life decisions and DMC in specific subgroups such as cancer or dementia patients [2,3,5-9]. Yet, little is known about the number of older people experiencing limitations in DMC in the general population, and how it affects end-of-life care, communication and use of ADs.

Therefore, we aim to explore how many older people develop limited DMC and how long before death these older people develop limited DMC. Second, we aim to describe the potential differences in background characteristics, the patient’s satisfaction with communication about care (according to their relative), and relatives’ satisfaction with communication about care between older people with full DMC until death and older people with limited DMC. In this, we also explore whether this is different for people who have limited DMC for a longer or shorter period before death. Third, we want to describe the experiences with use of ADs for people who had an AD and limited DMC for shorter or longer period before death, according to their relatives. Finally, we study whether the relatives of people with and without an appointed proxy AD differed in terms of the satisfaction with the role of the proxy in the decision-making process.

## Methods

### Study design and sample

Our sample consisted of deceased participants of two cohort studies. A representative sample of the Longitudinal Aging Study Amsterdam (LASA), and a purposive sample of the advance directive cohort study (ADC). The cohort members died between 2006–2009. As LASA is a cohort of older people, and the youngest deceased participant of the LASA cohort was 57, we selected deceased ADC participants from aged 57 years and over (omitting 9 younger members). Data about the last three months of life of the deceased people were collected using written questionnaires that were sent to a close relative. For the deceased sample members we checked who had given permission to contact a named relative after their death; in both cohorts this was asked when starting the cohort. If this permission was given, we sent a letter to the relative to ask whether they were willing to participate in the study via an answering card. The data collection took place in 2009–2010. The Medical Ethical Committee of the VU University Medical Center approved the study design. The samples included all deaths, including sudden deaths.

### LASA sample members

LASA is based on a nationally representative sample of older adults aged 55–85 years, stratified by age and gender and drawn from three regions in the Netherlands. These regions reflect the national distribution of urbanisation and population density. In 1992–1993 the LASA sample was recruited and a total of 3107 subjects were enrolled. Data collection takes place every three years. An additional cohort was recruited from the sampling frame exactly ten years after the first cycle of the cohort (n=1002 enrolled in 2002–2003). The sampling, data-collection and response rates of the LASA have been described more extensively elsewhere [10]**.** There were 311 participants in the LASA cohort who had died between 2006–2009 and had previously given permission to contact a proxy. Of them, 284 proxies were approached, as 27 proxies could not be found. A total of 168 proxies completed the questionnaire (59%), 69 proxies (25%) did not respond, and 47 proxies (17%) did not want to participate. The proxies of the deceased LASA members were predominantly a child of the deceased member (83%), followed by the partner (7%), another family member (5%), and non-relative (e.g. a friend) (5%).

### ADC sample members

In the Netherlands, two associations provide the most common types of ADs ‘Right to Die-NL’ (NVVE in Dutch) and Dutch Patient Association (NPV in Dutch). The NVVE provides four types of standard Ads: 1. a refusal-of-treatment document (ROTD) states in what situations a person does not want to receive life-prolonging treatment; 2. a do-not-resuscitate order (DNR); 3. a document in which somebody can appoint a health care proxy (proxy AD); and 4. An advance euthanasia directive (AED) in which a person can state in which situations he or she would wish life to be ended. As with oral euthanasia requests, it does not have to be granted by the physician and a physician is only allowed to grant when the criteria for due care are met. The NPV is a Christian oriented patient organization and provides a ‘will-to-live statement’ in which a person declares that he or she prefers to receive proper care, meaning no excessive, medically useless treatments at the end of life. In addition, it states that the person in question does not want active ending of life.

The advance directive cohort is based on a sample of 5561 people who have requested an AD from the Right to Die-NL (NVVE), and 1261 people who have formulated an AD through the Christian Dutch Patient Association (NPV). The first measurement was in 2005 with follow-ups every one and a half years. The entire design of the ADC study is described more extensively elsewhere [11]. Of the ADC participants, 263 died between 2006–2009 and had given permission to contact a proxy. Of them, 256 proxies were approached (NVVE n=232, NPV n=24), as 7 proxies could not be reached (NVVE n=6, NPV n=1). A total of 184 (NVVE n=167, NPV n=17) proxies completed the questionnaire (72%), 52 proxies (20%) did not respond, and 20 proxies (8%) were not willing to participate. The proxies of the deceased ADC members were a child of the deceased member (53%), followed by the partner (39%), and another family member (6%), and a non-relative (2%). The relationship between respondent and deceased differed significantly between the LASA and ADC cohort.

### Measures

All data were derived from the questionnaires the proxies of deceased LASA and ADC members had filled in. The proxies completed a questionnaire that consisted of structured questions including age, gender, DMC, possession of an AD, received care, contact about and satisfaction with care, communication about and influence of ADs on care, the role of the proxy in decision-making, and cause and place of death. Decision-making capacity was asked by the question ‘Until what moment was the deceased sample member capable of making end-of-life decisions’, with the response categories ‘until the moment of death, minutes before death, hours before death, days before death, a week before death, several weeks before death, a month before death, or more than a month before death’. The proxies were asked if the deceased sample member had formulated an AD (answered by yes, no, or do not know), and if so, which AD was formulated (one or more answers could be ticked in response categories appointed health care proxy, advance euthanasia directive, refusal of treatment document, do-not-resuscitate order, will-to-live statement). The questions about contact and satisfaction (concerning care (aim 2), the use of ADs (aim 3 and 4), role of the proxy (aim 4)) were distinguished in two types of questions: the proxies were asked to provide their perceived view of the decedents’ contact and satisfaction as well as the proxies’ own contact and satisfaction with the person’s care. With regard to the care being according to patient preferences, the proxies were asked to assess if the care received by the person was in accordance with the preferences of the person (yes, partly, no, unknown).

### Analyses

**Figure 1:**
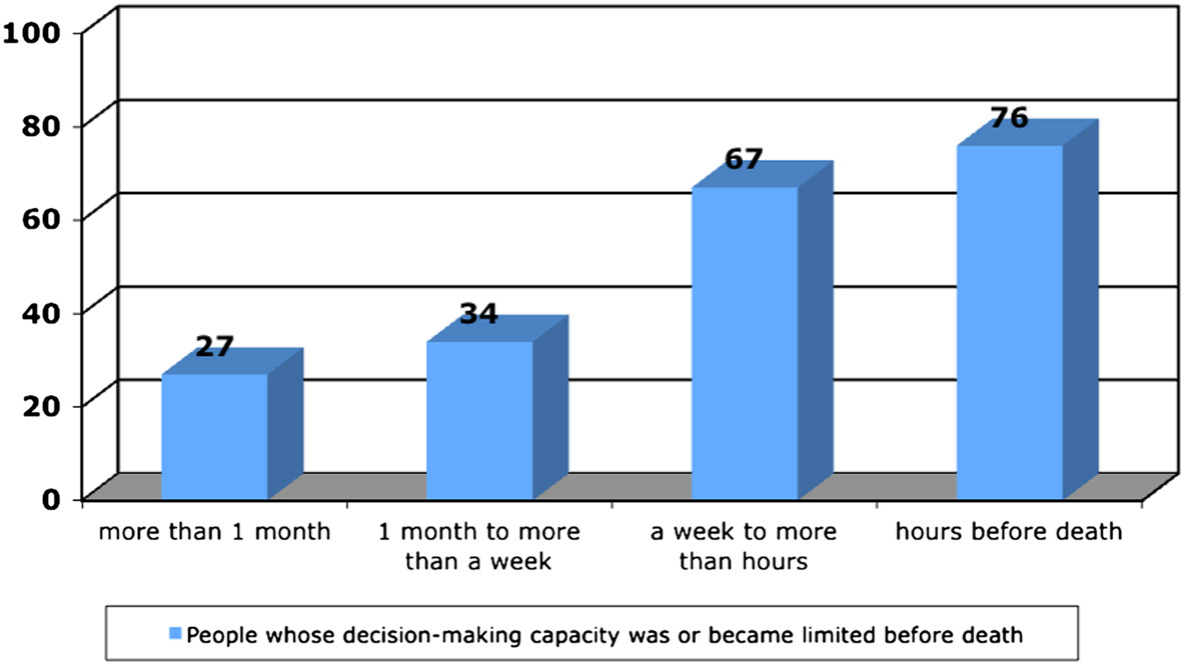
Period of limited decision-making capacity before death (LASA-sample, rounded percentages, n=165).

For describing the period of limited DMC of Dutch older people (aim 1 and 2), we used the deceased LASA sample members, since this sample is representative for the Netherlands. We included 165 of the 168 cases for which the question on limited DMC was filled in by the proxy. For describing background characteristics of deceased LASA sample members we recoded the variable on DMC in three categories: full DMC until death included ‘until a the moment of death’ and ‘minutes before death’; limited DMC before death a week or less before death, included ‘hours before death’, ‘days before death’ and ‘a week before death’; and limited DMC more than a week before death included ‘several weeks before death’, ‘a month before death’, and ‘more than a month before death’. We selected these categories as during preliminary analyses a shift of limited DMC was found especially during the last week of life (see also Figure 1). Differences between groups were tested with chi-square tests, using a level of significance of p < 0.05.

For the study aims that focused on (proxy) ADs, we used the cases from the LASA cohort and in addition we included people from the ADC cohort. Purpose for this was to have a larger number of people with a (proxy) AD. For describing experiences with ADs (aim 3), we selected people with an AD who had limited DMC before death (n=120). In the analyses we compared people with limited DMC for a week or less before death with people who had limited DMC more than a week before death. For studying the influence of a proxy AD (aim 4) we selected all people with limited DMC before death (n=213) and compared the group with a proxy AD with the group without a proxy AD. Differences between groups were tested with chi-square tests, using a level of significance of p < 0.05.

## Results

### Limited DMC

Figure 1 displays the period of limited DMC before death in the LASA sample. While 27% of the people experienced limited DMC more than a month before death, this percentage especially increased up to 67% during the last week of life. Only 24% remained lucid until shortly before death.

### DMC and patient and care characteristics

Older people with full DMC, limited DMC for a week or less, or limited DMC for more than a week before death did not differ in age, gender, and having an AD (Table 1). Both for people with and without limited DMC 17% had an AD, of which only 3% of people with full DMC and 1% of people with limited DMC had formulated an appointed proxy AD.

**Table 1:** Patient and care characteristics and decision-making capacity (LASA-sample, n=165, rounded percentages)*

	**Full DMC**	**Limited DMC before death**		
		**total**	**more than a week before death**	**a week or less before death**
	**n=39 (%)**	**n=126 (%)**	**n=56 (%)**	**n=70 (%)**
**Reason of incompetence**^**†**^	n.a			
Unconscious/coma^**‡**^		32	8	52
Dazed		32	23	40
Demented^**‡**^		27	59	0
Confused		19	26	12
Symptom burden		4	4	6
Not able/willing to think of end of life		4	4	5
Communication difficult		1	2	0
Stroke		1	2	0
Other		3	6	2
**Age**				
<70	15	14	7	19
70-85	51	43	43	43
>85	33	44	50	39
**Male**	59	47	43	50
**Cause of death**^**‡ ¶**^				
Cancer	16	25	13	28
Old age	11	23	36	13
Heart disease	32	12	13	12
Stroke	11	10	2	16
Lung disease (asthma/CARA/COPD)	8	10	4	15
Dementia	0	10	21	0
Other	22	11	13	10
**Advance Directive (AD)**	17	17	15	19
**Type of AD**^**†**^				
Advance euthanasia directive	6	8	11	6
Do-not-resuscitate order	6	6	2	8
Appointed proxy	3	1	2	0
Refusal of treatment document	3	3	2	3
Will-to-live statement	3	2	0	3
**Type of care during the last 3 months of life**				
District nurse	39	45	39	49
Informal home care	53	36	27	43
Formal home care	36	34	25	42
Nursing home care ^**¶**^	3	29	7	15
Volunteer help	10	9	13	6
**Main attending physician in last three months**^**‡ ¶**^				
General practitioner	86	59	45	70
Specialist elderly care	3	29	7	17
Medical specialist	11	13	48	13
**Hospitalization in last month of life**^**‡**^	37	36	22	49
**Place of death**^**‡ ¶**^				
Hospital	28	28	13	41
Own home	45	26	23	28
Nursing home	8	27	52	6
Residential home	17	15	11	22
Hospice	3	3	2	3

^*^Missing observations were less than 5% of the total n, except for the group ‘full DMC until death’, between 1 and 9 missing observations.

^**†**^More than one answer could be given.

^**‡**^Difference between the groups ‘limited DMC more than a week before death’ and ‘limited DMC a week or less before death’ significant (p<0.05).

^**¶**^Difference between groups ‘full DMC until death’ and ‘limited DMC before death significant’ significant (p<0.05).

For people with limited DMC a week or less before death, the limitation in DMC was mostly due to being unconscious or comatose state (52%). In people with limited DMC more than a week before death, dementia was the most common cause of limited DMC (59%). People who had full DMC until death were more often diagnosed with a heart disease than people with limited DMC (32% versus 12%). The two groups of people with limited DMC significantly differed in diagnosis: for people with limited DMC a week or less before death the diagnosis was more often cancer (28% versus 13%) and less often old age (13% versus 36%) or dementia (0% versus 21%) than for people with limited DMC more than a week. While there was no difference between hospitalization in the last month of life between people with full DMC and limited DMC overall (full DMC 37%, limited DMC 36%), people with limited DMC for more than a week were hospitalized in the last week of life less often than people with limited DMC a week or less before death (22% versus 49%).

People with full DMC mostly died in their own home: in 45% of cases compared to 26% of people with limited DMC dying at home. The two groups of people with limited DMC significantly differed in place of death: people with limited DMC a week or less before death more often died in hospital (41% versus 13%) and less often in a nursing home (6% versus 52%) than people with limited DMC for more than a week before death.

### DMC and (satisfaction with) communication about care

As shown in Table 2, according to the relatives of the deceased, in more than half of the cases, care received was in general in accordance with the patient’s preferences both for people with full and people with limited DMC (both 71%). Looking within the group of people within limited DMC, people with limited DMC for more than a week significantly less often received care according to their preferences (60%) than people with limited DMC for a week (81%).

**Table 2:** Communication of physician with older people and their relatives and decision-making capacity before death (LASA-sample, n=165, rounded percentages)*

	**Full DMC**	**Limited DMC before death**		
		**total**	**more than a week before death**	**a week or less before death**
	**n=39**	**n=126**	**n=56**	**n=70**
	**(%)**	**(%)**	**(%)**	**(%)**
**Received care in general in accordance with preferences of patient**^**†**^				
Yes	71	71	60	81
Partly	8	12	13	10
No	3	3	7	0
Don’t know	18	14	20	9
**Physician communicated understandably concerning treatment choices**^**† ‡**^				
Yes, to patient and relative	56	58	49	65
Yes, only to patient	6	5	2	8
Yes, only to relative	0	20	31	11
No	19	9	13	6
Don’t know	19	8	6	11
**Patient satisfaction with contact between physician and patient**^**†**^				
(Very) satisfied	71	67	54	77
Neutral	18	14	13	15
(Very) unsatisfied	0	2	4	0
Don’t know	12	18	30	9
**Satisfaction of relative with contact between physician and patient**				
(Very) satisfied	79	75	71	79
Neutral	12	18	20	16
(Very) unsatisfied	9	7	9	5
**Satisfaction of relative with his/her contact with physician**				
(Very) satisfied	81	73	69	76
Neutral	10	18	20	16
(Very) unsatisfied	10	9	11	8

^*^Missing observations were less than 5% of the total n, except for the group ‘full DMC until death’, between 1 and 9 missing observations.

^**†**^Difference between the groups ‘limited DMC more than a week before death’ and ‘limited DMC a week or less before death’ significant (p < 0.05).

^**‡**^Difference between groups ‘full DMC until death’ and ‘limited DMC before death significant’ significant (p < 0.05).

While in all groups the physician most frequently communicated treatment choices to patient and relative (between 49% and 65%), they most frequently discussed this only with the relative in people with limited DMC for a week or more (31%). Relatives, when asked about their own experiences, were in large majority satisfied with contact between physician and patient, and between him or herself and the physician; no significant differences between the different groups were found. The percentage of relatives being (very) unsatisfied ranged from 5% to 11%.

### DMC and the role of ADs in communication

For people with limited DMC and with an AD (Table 3), the majority of the physicians were aware of the AD (83% and 87%) and the content of the AD (fully aware 82% and 76%), according to their relatives. However, the two groups of different periods of limited DMC differed significantly concerning the time at which the physician had been informed about the patient having an AD: for people with limited DMC more than a week before death the physician had more often been informed before the illness (83% versus 52%) than people with limited DMC for a week or less. The relatives of people with limited DMC during the last week of life reported more often that the physician had not discussed the AD during the last week of life than relatives of patients with limited DMC for a week or more (41% versus 21%). Relatives of people with limited DMC a week or less before death more often said they perceived their own communication with physicians was ‘good’ (63% versus 48%), compared to the relatives of people with limited DMC more than a week before death. In both groups over half of the relatives considered the AD to be of additional value (54% and 65%). In both groups about one third of relatives thought the ADs had not had influence of care, mostly because the AD did not relate that the patient’s situation (15% and 17%), but also because the physician did not want to cooperate (11% and 2%).

**Table 3:** Experiences with the use of ADs of older people who had and AD and with limited decision-making capacity before death (n = 120, rounded percentages)^*^

	**Older people with an AD who had limited decision-making capacity more than a week before death**	**Older people with an AD who had limited decision-making capacity a week or less before death**
	**n=36**	**n=84**
	**(%)**	**(%)**
**Responsible physician was aware of the existence of the AD(s)**		
Yes	83	87
No	6	2
Don’t know	11	11
**Responsible physician was aware of the content of the AD(s)**		
Yes, fully	82	76
Yes, globally	14	21
No	4	3
**Time of physician being informed about the AD(s)**^**‡**^		
Before illness	83	52
During illness	13	47
Close to the moment of death	4	2
**Discussion about AD in the last week of life with physician**^**‡**^		
Yes, with patient	38	32
Yes, with proxy	33	17
No	21	41
Don’t know	8	10
**Relative’s perspective on communication process about AD(s)**^**‡**^		
Good	48	63
Not good, not bad	12	11
Bad	32	11
No communication	8	10
Don’t know	0	5
**Relative’s perspective on additional value of AD(s)**		
Additional	54	65
Neutral	17	18
Not additional	29	17
**Relative’s perspective on influence of AD(s) on care**		
AD determined decisions	12	22
AD was (very) influential	31	28
AD had little influence	27	15
AD had no influence, because:	31	35
- did not relate to patient’s situation	15	17
- the physician did not want to cooperate	11	2
- the AD was signed too long ago	4	7

^*^Missings in all groups of all variables were less than 5% of the total N; 101 respondents of ADC sample and 19 respondents of LASA sample.

^**‡**^Difference between the two groups significant (p < 0.05).

### Proxy and having an appointed proxy AD

According to the relatives, approximately two-thirds of the people with and without an appointed proxy AD (Table 4), in general received care in accordance with the patient’s preferences (both 67%). However, for a small percentage of people without a proxy AD it was not known whether care was in accordance with the patient’s preferences (11%, with AD 0%). No significant differences were found between both groups concerning being satisfied with the influence of patient and proxy in decision-making. The majority of proxies of people both with and without proxy AD reported to be (very) satisfied.

**Table 4:** Role of the relative in decision-making for older people with limited decision-making capacity before death and with or without an appointed proxy AD (n=213, rounded percentages)

	**Older people without an appointed proxy AD**	**Older people with an appointed proxy AD**
	n=163*	n=50*
	**(%)**	**(%)**
**Received care in accordance with preferences of patient**^**†**^		
Yes	67	67
Partly	17	20
No	6	12
Don’t know	11	0
**Patient’s satisfaction with contact between physician and patient**		
Satisfied	68	68
Neutral	22	19
Not satisfied	11	13
Don’t know		
**What influence in decision-making did relative have during the last 3 months**		
Determined decisions	6	10
Influential	45	45
Not very influential	17	20
Not influential	12	12
No decisions had to be made	20	12
**Satisfaction of relative with their influence in decision-making**		
Satisfied	70	70
Neutral	23	16
Not satisfied	7	14
**Satisfaction of relative with end-of-life care and consideration of preferences**		
Satisfied	78	74
Neutral	17	13
Not satisfied	5	13

^*^Missings in all groups of all variables were less than 5% of the total N; people without an appointed proxy AD: 54 respondents of ADC sample and 109 respondents of LASA sample; people with a proxy AD: 49 respondents of ADC sample and 1 respondents of LASA sample.

^†^Difference between the two groups significant (p <0. 05).

## Discussion

While about a quarter of older people has limited DMC for more than a month before death, there is especially an increase in limited DMC in the last week of life. In the last week two thirds of older people have limited DMC; about a quarter remains lucid until death. Limited DMC a week or less before death is mostly due to reduced consciousness or coma, while limited DMC more than a week before death is predominantly due to dementia. People who had full DMC until death died significantly more often of a heart disease than people with limited DMC before death. The care received was in accordance with the decedent’s preferences for the majority of older people although less often for people who had limited DMC for more than a week. The majority of the relatives were satisfied with the communication between physician and the patient and them, regardless of DMC of the patient. Of older people with limited DMC and an AD, relatives described the communication process about ADs more frequently as good for patients with limited DMC for a week or less than for patients with limited DMC for more than a week. A small majority of relatives indicated that the AD had been of additional value. Finally, no differences were found in the role of the relative and the satisfaction with this role between people with and without a proxy AD.

### Strengths and limitations

A strength of our study is the combination of two cohorts with different strengths. The LASA cohort is representative for the Dutch older population allowing us to study the first two aims of getting insight into frequency and circumstances of limited DMC in the last phase of life of older people. Since ADs do not frequently occur in the Dutch older population, the ADC cohort allowed us to study ADs in relation to communication about care in people with limited DMC. A limitation of this study is that DMC was examined with a single question. Also, the sample was constituted of all deaths, including sudden death without an end-of-life phase and decision-process. However, these deaths especially occurred in the group with full DMC. That data were collected through proxies of died people brings two types of bias. First is potential recall bias due to the retrospective reporting: the cohort members deceased between half a year and three and a half years before the proxies entered the study [12]. However, it is likely that proxies remember circumstances around the death of a relative. Second, it is known that proxies are not always accurate in assessing the patient’s own views. Yet, literature indicates that proxies are more likely to give accurate information when it concerns more factual information such as care characteristics of a deceased relative, while less factual information is more likely to be assessed less accurate by proxies [12,13]. Of course, the experiences of the relatives themselves concern an important focus of this study, and for that part the proxies are the most suitable respondents Finally, a limitation of the study is that reports about overall satisfaction of patients in care can be limited and optimistic, although it has been found that asking about satisfaction on specific aspects of care (e.g. contact) can be more meaningful [14].

### DMC and background characteristics

DMC may not be limited during a large part of an illness trajectory, yet, towards the end of life a considerable number of people develop limited DMC, especially during the last week before death. People usually associate limited DMC with dementia. However, limited DMC can also develop in people with chronic physical illnesses [9]. We did find that the reason of limited DMC is related to the length of limited DMC, with people with illnesses related to old age such as dementia generally having limited DMC than people with other chronic diseases such as cancer. These findings are in line with the chronic illness trajectories described by Murray et al. [15]. Of course, people can also die suddenly, e.g. through a heart attack or fatal stroke; thus without an illness trajectory [16]. This group is generally lucid until death.

### DMC and (satisfaction with) communication about care

The time period of limited DMC can affect continuing communication in end-of-life decision-making [2,9]. However, our findings showed that all groups of patients and their relatives were in majority satisfied with their contact with the physician. It has been reported that information giving by a physician is the most important factor in care satisfaction for older people [17]. Even though people had limited DMC, the physician in many cases communicated about treatment choices with the patients themselves, and although people had full DMC, the physician frequently also communicated treatment choices with relatives. These findings underline that physicians generally approach both patient and relatives during the decision-making process. This is probably related to the fact that the decline in patients’ DMC is a process in which it is difficult to demarcate the moment that DMC becomes limited [2]. It might also be related to the physicians recognizing that it might be beneficial to include both patients and relatives in discussing care as the needs of the family may exceed those of the patient [18]. A timely start with advanced care planning by the physician and patient with full DMC is recommended to encompass diverse perceptions of autonomy and DMC [8] and advocate for the patient’s quality of life [19]. Of course in this one has to realize that people may theoretically have full DMC and the mental abilities to discuss treatment, while they may emotionally not be ready to accept the course of their illness and choose what treatment options to pursue in a particular situation [9,20]. This is in line with our finding of a small group of older people who had limited DMC because they were not able or willing to think of the end of life.

### DMC and the role of ADs

While in general a majority of relatives, thus also a large group of relatives of older people without AD, indicated care being according to the patient preferences and being satisfied with the communication with the physician, the relatives of persons with an AD have positive experiences with the use of the AD. In older people with limited DMC for more than a week the AD was discussed with the patient or relative in the last week of life of the patient in three quarter of cases; in older people with limited DMC in the last week or less this was done in about half of the cases. Over half of the relatives considered the AD of additional value, while at the same time only a small group considered that the AD determined decisions. This might indicate that relatives also see the role of an AD more as an aid in communication on end-of-life care and treatment, as some authors have suggested [21,22]. However, it would be desirable for future research to get more in-depth insight in the ways in which relatives experience the additional value of ADs.

It is remarkable that the satisfaction with the decision-making process of people with and without an appointed proxy AD was similar, as reported by relatives. This may be influenced by the fact that when patients develop limited DMC, physicians generally discuss treatment choices with relatives, a result supported by the results of a previous study [23]. Therefore, it seems that an appointed proxy AD is not necessarily needed to experience a satisfying decision-making process in which relatives are involved. However, the beneficial value of this AD might be the increasing of awareness of preferences of older people. Among relatives of older people without an appointed proxy AD, it was for a small percentage of people not known if care was in accordance with preferences of the deceased.

## Conclusions

In conclusion, a considerable number of people developed limited DMC, especially during the last week of life a. Although people may fear becoming limited in DMC, our study suggests that becoming limited in DMC does not affect satisfaction of relatives in care at the end of life. Our study does not provide strong evidence that (proxy) ADs have much influence in the last phase of life. However, relatives of people with an AD in majority had positive experience with the use of the AD in the last phase of life. It can probably best be seen as a tool that can be useful in advance care planning.

## Competing interests

The authors declare that there is no conflict of interest.

## Author’s contributions

Author Contributions: PJK: data collection, analysis and interpretation of data, and preparation and drafting of the manuscript. BDOP, DJHD and HRWP: study concept and design, analysis and interpretation of data, and preparation and drafting of the manuscript. All authors made substantial contributions to the article and approved the final content.

## Pre-publication history

The pre-publication history for this paper can be accessed here:

http://www.biomedcentral.com/1472-684X/12/1/prepub
